# Synthesis, characterization, molecular docking, analgesic, antiplatelet and anticoagulant effects of dibenzylidene ketone derivatives

**DOI:** 10.1186/s13065-018-0507-1

**Published:** 2018-12-06

**Authors:** Tauqeer Ahmed, Arif-ullah Khan, Muzaffar Abbass, Edson Rodrigues Filho, Zia Ud Din, Aslam Khan

**Affiliations:** 1 Riphah Institute of Pharmaceutical Sciences, Riphah International University, Islamabad, Pakistan; 2 LaBioMMi, Department of Chemistry, Federal University of São Carlos, CP 676, São Carlos, SP 13565-905 Brazil; 3 Department of Chemistry, Woman University Swabi, GulooDehri, Topi Road, Swabi, KP 23340 Pakistan; 4 Basic Sciences Department, College of Science and Health Professions-(COSHP-J), King Saud bin Abdulaziz University for Health Sciences, Jeddah, Saudi Arabia; 5 Present Address: Department of Pharmacy, Capital University of Science and Technology, Islamabad, Pakistan

**Keywords:** Dibenzylidene ketone derivatives, Computational studies, Analgesic, Antiplatelet, Anticoagulant, Arachidonic acid

## Abstract

In this study dibenzylidene ketone derivatives (2*E*,5*E*)-2-(4-methoxybenzylidene)-5-(4-nitrobenzylidene) cyclopentanone (AK-1a) and (1*E*,4*E*)-4-(4-nitrobenzylidene)-1-(4-nitrophenyl) oct-1-en-3-one (AK-2a) were newly synthesized, inspired from curcuminoids natural origin. Novel scheme was used for synthesis of AK-1a and AK-2a. The synthesized compounds were characterized by spectroscopic techniques. AK-1a and AK-2a showed high computational affinities (E-value > − 9.0 kcal/mol) against cyclooxygenase-1, cyclooxygenase-2, proteinase-activated receptor 1 and vitamin K epoxide reductase. AK-1a and AK-2a showed moderate docking affinities (E-value > − 8.0 kcal/mol) against mu receptor, kappa receptor, delta receptor, human capsaicin receptor, glycoprotein IIb/IIIa, prostacyclin receptor I_2_, antithrombin-III, factor-II and factor-X. AK-1a and AK-2a showed lower affinities (E-value > − 7.0 kcal/mol) against purinoceptor-3, glycoprotein-VI and purinergic receptor P_2_Y_12_. In analgesic activity, AK-1a and AK-2a decreased numbers of acetic acid-induced writhes (*P *< 0.001 vs. saline group) in mice. AK-1a and AK-2a significantly prolonged the latency time of mice (*P *< 0.05, *P *< 0.01 and *P *< 0.001 vs. saline group) in hotplate assay. AK-1a and AK-2a inhibited arachidonic acid and adenosine diphosphate induced platelet aggregation with IC_50_ values of 65.2, 37.7, 750.4 and 422 µM respectively. At 30, 100, 300 and 1000 µM concentrations, AK-1a and AK-2a increased plasma recalcification time (*P *< 0.001 and *P *< 0.001 vs. saline group) respectively. At 100, 300 and 1000 µg/kg doses, AK-1a and AK-2a effectively prolonged bleeding time (*P *< 0.001 and *P *< 0.01 vs. saline group) respectively. Thus in-silico, in-vitro and in-vivo investigation of AK-1a and AK-2a reports their analgesic, antiplatelet and anticoagulant actions.

## Introduction

Pain is an unfavorable sensory and emotional experience that is associated with the potential tissue damage and explained in terms of such damage [1]. Noxious effects such as ulceration, gastrointestinal bleeding by non-steroidal anti-inflammatory drugs and drowsiness, nausea and tolerance by opiates usage limits their use in management of pain [2]. Platelets play vital role in a complex processes which are involved in haemostasis and thrombosis [3]. The most common cause of peripheral artery diseases (PAD) is atherosclerosis and such patients have more chance of myocardial infarction, stroke or death with cardiovascular events and it is 3:1 in comparison to persons without PAD [4]. Antiplatelet agents are used in management of arterial thrombosis. Moreover, anticoagulants inhibit proteases in coagulation cascade [5]. Interference in natural balance among pro-coagulant and anticoagulant due to genetic or any other acquired factors may results in bleeding or thrombotic disorders. Thrombin is a key enzyme of coagulation cascade which has many significant biological functions including platelet activation, fibrinogen conversion to fibrin network and feedback amplification of coagulation. Different tissue factors are involved in thrombus formation in order to prevent heamorrhage [6]. Coagulation cascade involves intrinsic and extrinsic pathways [7]. The former has a role in the growth and maintenance of fibrin while the later plays its role in the initiation of fibrin formation. Extrinsic pathway requires tissue factors for its activation which after vascular injury becomes exposed to the blood which ultimately results in thrombin activation [8]. Among antiplatelet agent and anticoagulant drugs which are available commercially, for thrombotic disorders, these agents are associated with certain limitations and side effects [9]. Chemically curcumin is 1,7-bis (4-hydroxy-3-methoxyphenyl)-1,6-heptadiene-3,5-dione. It is a yellow-orange colored pigment which is derived from the rhizome of *Curcuma longa* [10]. The plant has a wide spectrum of pharmacological properties and traditionally it has been used for many ailments since centuries [11]. The reported activities of curcumin are antioxidant, anti-inflammatory, antitumor, antibacterial, antifungal and antiviral [10]. Curcumin also showed inhibition in platelet aggregation and antithrombotic effects [12, 13]. Concerning structural aspects, dibenzylidene ketone moieties are considered curcumin analogues, which are compounds of great importance. Structurally, curcuminoids contains two aryl rings connected at the ends of a C_7_ carbon-chain where a dienone composes an extended conjugated system. Dibenzylidene ketone derivatives also contain a dienone system connecting two aryl groups at the ends of a C_5_ carbon chain. Dienones are good Michel acceptors, allowing its reaction with important biomolecules interfering in biological processes. Previous reported activities of dibenzylidene ketone derivatives include antiparasitic activity, cytotoxicity, antimicrobial activity, analgesic activity [14–16]. Based on previous literature studies, two novel dibenzylidene ketone derivatives i.e. (2*E*,5*E*)-2-(4-methoxybenzylidene)-5-(4-nitrobenzylidene) cyclopentanone (AK-1a) and (1*E*,4*E*)-4-(4-nitrobenzylidene)-1-(4-nitrophenyl) oct-1-en-3-one (AK-2a) were synthesized and characterized. AK-1a and AK-2a were investigated for their analgesic, antiplatelet and anticoagulant effects using different pharmacological and computational assays.

## Materials and methods

### Chemicals

Adenosine diphosphate (ADP) and arachidonic acid (AA) were purchased from Chrono-Log association. Benzaldehyde, cyclopentanone, dimethyl sulfoxide, ethanol and methoxybenzaldehyde were purchased from Merck Millipore., Billerica, MA, USA. Aspirin, calcium chloride (CaCl_2_), diclofenac sodium, heparin, phosphate buffers solution (PBS) and sodium citrate were obtained from Sigma chemicals., Dt. Louis, MO, USA. The tramadol was acquired from Searle Karachi-Pakistan. All chemicals used were of analytical grade.

### Animals

Balb-C mice (25–30 g) of both gender were utilized for this study. All animals were housed according to the standard protocols 25 ± 2 °C, 12 h duration of natural light and dark cycle. Healthy diet was given to mice and water ad libitum. The study was performed in accordance with protocols of Institute of Laboratory Animal Resources, Commission on Life Sciences University, National Research Council (1996) and approved by Riphah Institute of Pharmaceutical Sciences (RIPS) Ethical Committee (Reference No: REC/RIPS/2016/009).

### Synthesis of AK-1a and AK-2a

**Fig. 1:**
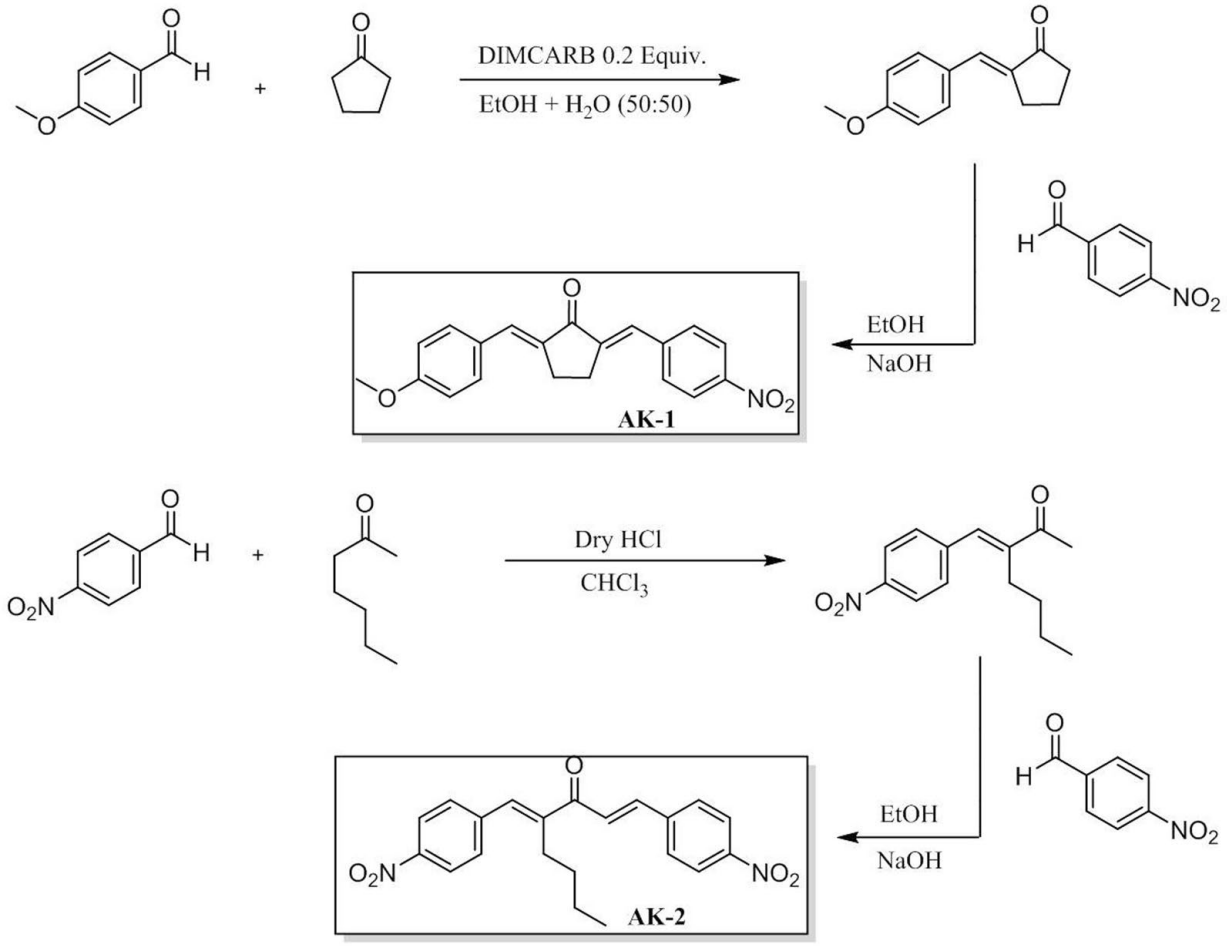
Chemical structure and synthesis of (2*E*,5*E*)-2-(4-methoxybenzylidene)-5-(4-nitrobenzylidene) cyclopentanone (AK-1a) and (1*E*,4*E*)-4-(4-nitrobenzylidene)-1-(4-nitrophenyl)oct-1-en-3-one (AK-2a)

**Fig. 2:**
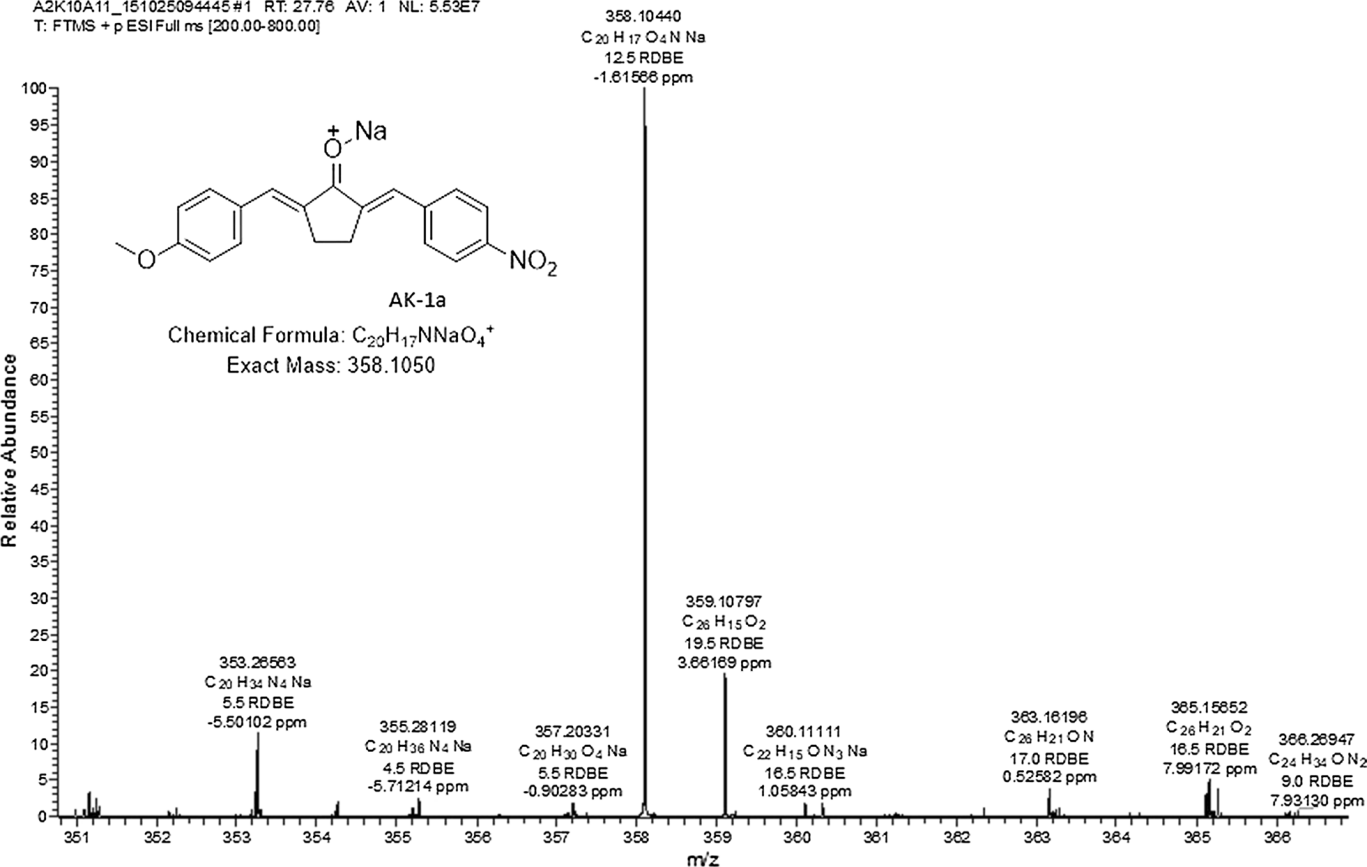
Represents Fourier transform mass spectrometry (FTMS) of (2*E*,5*E*)-2-(4-methoxybenzylidene)-5-(4-nitrobenzylidene) cyclopentanone (AK-1a)

**Fig. 3:**
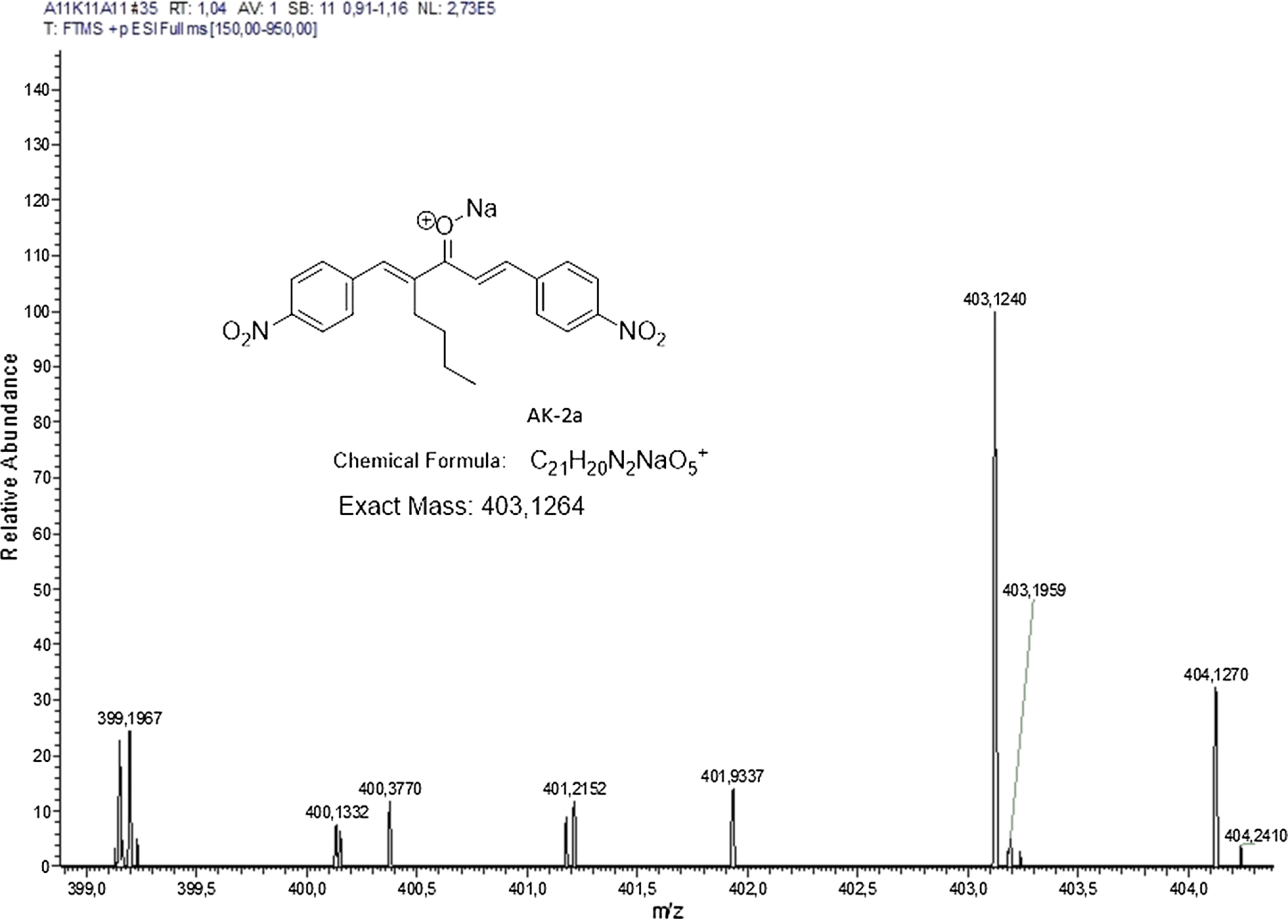
Represents Fourier transform mass spectrometry (FTMS) of (1E,4*E*)-4-(4-nitrobenzylidene)-1-(4-nitrophenyl)oct-1-en-3-one (AK-2a)

Novel way of synthesis was carried out. The monoarylidene derivative was synthesized by the reaction of cyclopentanone with *p*-methoxy benzaldehyde. DIMCARB was utilized as a catalyst in this reaction. DIMCARB was used in catalytic amount to obtain selective monoarylidene cyclic derivative in a green solvent (EtOH:H_2_O), further second step leads to get an unsymmetrical bis-(arylmethylidene)-cycloalkanones. The synthesis of compound was carried out at room temperature from the reaction of intermediate 1 with *p*-nitro benzaldehyde. The scheme of the synthesized compound along with its structure is shown in Fig. 1. Chemical characterization was carried out based on the analysis of spectroscopic data. Fourier transform mass spectrometry (FTMS) of AK-1a as shown in Fig. 2. Synthesis of AK-2a was carried in a two-step reaction. In the first step, cycloalkanone was reacted with an aldehyde in a DIMCARB catalysed reaction, while in the second step monoarylidene derivative was reacted with the aldehyde through knoevenagel condensation to get the required product. DIMCARB can be recovered by distillative dissociation–reassociation process in a vacuum or under an atmosphere of CO_2_. The 2-heptanone was reacted with *p*-nitro benzaldehyde in an acidic medium to get intermediate, and then intermediate yield AK-2a. The scheme of the novel synthesized compound AK-2a along with its structure is shown in Fig. 1. Chemical characterization was carried out based on the analysis of spectroscopic data. Fourier transform mass spectrometry (FTMS) of AK-2a as shown in Fig. 3.

### Spectral analysis

#### AK-1a

Percent yield: 84. Decompose at: 96–98 °C. ^1^H NMR (400 MHz, CDCl_3_) δ 7.57 (d, J = 8.8 Hz, 3H), 7.52 (d, J = 8.4 Hz, 3H), 7.40 (d, J = 8.6 Hz, 2H), 6.97 (d, J = 8.9 Hz, 2H), 3.86 (s, 3H), 3.08 (s, 4H). ^13^C NMR (101 MHz, CDCl_3_) δ 196.21 (1C), 160.90 (1C), 138.27 (1C), 135.28 (1C), 134.71 (1C), 134.32 (1C), 132.73 (2C), 131.93 (2C), 129.16 (2C), 128.70 (1C), 114.51 (2C), 77.48 (1C), 76.84 (1C), 55.54 (1C), 26.60 (2C). HRMS ESI(+): calcd for C_20_H_17_NNaO_4_^+^ (M + Na) 358.1050, found 358.1044.

#### AK-2a

Percent yield: 80. m.p: 180.5–181.5 °C. ^1^H NMR (400 MHz, CDCl_3_) δ 8.28 (dd, J = 8.5, 7.7 Hz, 4H), 7.80–7.70 (m, 3H), 7.56 (d, J = 8.7 Hz, 2H), 7.49 (s, 1H), 7.43 (d, J = 15.7 Hz, 1H), 2.65–2.58 (m, 2H), 1.53–1.42 (m, 2H), 1.37 (dd, J = 14.7, 7.2 Hz, 2H), 0.91 (t, J = 7.2 Hz, 3H). ^13^C NMR (101 MHz, CDCl_3_) δ 191.74 (1C), 148.73 (1C), 147.58 (1C), 146.65 (1C), 142.33 (1C), 141.43 (1C), 141.07 (1C), 135.88 (1C), 129.97 (2C), 129.03 (2C), 125.92 (1C), 124.38 (2C), 124.01 (2C), 31.22 (1C), 27.24 (1C), 23.03 (1C), 13.94 (1C). HRMS ESI(+): calcd for C_21_H_20_N_2_ NaO_5_^+^ (M + Na) 403.1264, found 403.1240.

### In-silico study

Molecular docking is an informative tool which is used to investigate the affinity between ligand and protein targets. We used Auto Dock Vina program for docking study through PyRx [17, 18]. Affinity of best docked pose of ligand and protein target complex was determined by E-value (kcal/mol). It provides prediction of binding free energy and binding constant for docked ligands [19]. 3D-structures of test compounds (AK-1a and AK-2a) were prepared in discovery studio visualiser (DSV) and saved as PDB format. 3D-structures of target proteins were taken from http://www.rcsb.org/pdb/home/home.do. The target proteins involved in pain pathways are cyclooxygenase-1 (COX-1, PDB-ID: 3N8X), cyclooxygenase-2 (COX-2, PDB-ID: 1PXX), mu receptor (PDB-ID: 5C1M), kappa receptor (PDB-ID: 4DJH), delta receptor (PDB-ID: 4EJH), human capsaicin receptor (HCR, PDB-ID: 3J9J) and purinoceptor-3 (P2X3, PDB-ID: 5SVL). The target proteins involved in platelet aggregation are glycoprotein-IIb/IIIa (GP-IIb/IIIa, PDB-ID: 2VDM), glycoprotein-VI (GP-VI, PDB-ID: 2G17), purinergic receptor (P_2_Y_12_, PDB-ID: 4PXZ), prostacyclin receptor I_2_ (PG-I_2_, PDB-ID: 4F8K) and proteinase-activated receptor 1 (PAR-1, PDB-ID: 3VW7). The target proteins involved in blood coagulation process are antithrombin-III (AT-III, PDB-ID: 2B4X), factor-II (F-II, PDB-ID: 1KSN), factor-IX (F-IX, PDB-ID: 1XMN), factor-X (F-X, PDB-ID: 1RFN) and vitamin-K epoxide reductase (VKOR, PDB-ID: 3KP9). All the target proteins were then purified by removing ligands and other entities which might occupy nearby space using Biovia Discovery Studio Client 2016. The structures of standard drug molecules were downloaded from pubchem data base (https://pubchem.ncbi.nlm.nih.gov/search/). Reference analgesic drugs are aspirin (PubChem CID: 2244), morphine (PubChem CID: 5288826) and capsazepine (PubChem CID: 2733484). Standard antiplatelet drugs are aspirin (PubChem CID: 2244), tirofiban (PubChem CID: 60947), hinokitiol (PubChem CID: 3611), clopidogrel (PubChem CID: 10066813), beraprost (PubChem CID: 6917951) and vorapaxar (PubChem CID: 10077130). Reference anticoagulant drugs are heparin sulphate (PubChem CID: 53477714), apixaban (PubChem CID: 10182969), argatroban (PubChem CID: 92722), pegnivacogin (PubChem CID: 86278323) and warfarin (PubChem CID: 54678486). All these structures were downloaded in .xml format and converted to PDB format via Open Babel JUI software. PDB form of both ligand and standard as well as target proteins were converted to PDBQT via AutoDockTools (Version1.5.6 Sep_17_14) where add kollman charges and compute gastegier charges were added and Ad4 type was assigned. Both the test compounds along with protein targets in PDBQT form were loaded in software named as PyRx and then docked against the respective targets. Binding affinity was calculated and shown in kcal/mol. For post docking interaction Discovery studio visualizer was used for number of hydrogen bonds (classical and non-classical) and binding amino acid residues: alanine (ALA), asparagine (ASN), arginine (ARG), aspartic acid (ASP), cysteine (CYS), glutamine (GLN), glutamic acid (GLU), glycine (GLY), histidine (HIS), leucine (LEU), lysine (LYS), serine (SER), threonine (THR), tryptophan (TRP), tyrosine (TYR), valine (VAL) and phenylalanine (PHE) showed in the form 2D interaction.

### Analgesic models

The analgesic activity was carried out by using two standard protocols i.e. acetic acid-induced writhing test and hot plate test in order to evaluate the peripheral and central effects of analgesia.

#### Acetic acid-induced writhing test

Mice were divided into five different groups, having five mice in each. After 30 min writhing were induced by an IP injection of 0.1 mL of 0.7% (by volume) acetic acid solution [20]. Drug pretreatment times were chosen so that writhing was counted over a period of maximum analgesic activity. AK-1a and AK-2a in a dose-dependent manner (0.5–100 mg/kg) decreased acetic acid-induced writhes injected through intraperitoneal (IP) route. Perception of pain was recorded in the form of abdominal constrictions and stretches of the hind limb called as a writhe. Some mice showed half writhe. Two half writhes were considered as equal to one full writhe. The writhing episodes were recorded for 20 min. Control group was administered with normal saline (10 mL/kg). Diclofenac sodium was used as a positive control.

#### Hot plate test

The latency period of the test compounds were evaluated by hot plate assay according to the protocols as previously used with little modifications [21]. Mice were divided into five different groups, having five mice in each. The animals were placed individually on the hot plate (55 ± 2 °C) and the observations (jumping or licking paws) were recorded at 30, 60, 90 and 120 min. Normal saline (10 mL/kg) was given to control group, tramadol (30 mg/kg) was used as a positive control.

### Antiplatelet assay

Antiplatelet activity was performed to check whether the test compounds possess any effect on platelet aggregation. It was determined by whole-blood aggregometry method, which was performed by an impedance aggregometer (Model 591, Chrono-Log) as previously described [22]. Arterial or venous blood samples were collected from healthy volunteers in plastic tubes having 3.2% sodium citrate anticoagulant (9:1). Measurements were performed at 37 °C and 1200 rpm stirring speed. According to the manufacturer recommendations, 0.5 mL of citrated blood was diluted with same volume of normal saline (0.9%) which was prewarmed for 5 min at 37 °C. 30 µL, AK-1a and AK-2a at 1, 3, 10, 30, 100, 300, 1000 µM concentrations were also added to the tube. After placing the electrode, aggregation was induced by different agonists like AA (1.5 mM) and ADP (10 µM). Platelet aggregation response was continually monitored for 6 min as an electrical impedance in ohms. Then mean percent platelet inhibition was calculated. Aspirin was used as positive control.

### Anticoagulant activity

Anticoagulant activity of the test compounds were performed using following experiments.

#### Plasma recalcification time (PRT)

Anticoagulant potential of the test compounds were determined by PRT method [23]. The blood samples were obtained from healthy volunteers in tubes containing 3.8% sodium citrate (9:1) in order to prevent the clotting process. Centrifugation (15 min at rate 3000 rpm) was carried out in order to obtain platelet poor plasma. 0.2 mL plasma, 0.1 mL of different concentration of the test compounds (30, 100, 300 and 1000 μM) and 0.3 mL of CaCl_2_ (25 mM) were then added together in a clean fusion tube and incubated in a water bath at 37 °C. Heparin (440 μM) was used as positive control. The clotting time was recorded with a stopwatch by tilting the test tubes every 5 s.

#### Bleeding time (BT)

Anticoagulant potential of AK-1a and AK-2a was also determined by in-vivo tail BT method in mice [24]. AK-1a and AK-2a (100, 300 and 1000 μg/kg) were administered intravenously via tail vein of mice. After 10 min mice were anesthetized using diethyl ether and a sharp cut (3 mm) deep at tip of the tail were made. The tail was then immersed into PBS which was pre warmed to 37 °C. BT was recorded from the time when bleeding started till it stopped completely observation was made up-to 10 min. Heparin (40 μg/kg) was utilized as a positive control.

### Statistical analysis

Data were expressed as mean ± standard error of mean (SEM) and analyzed by using one-way analysis of variance (ANOVA), with post hoc Tukey’s test. Data were considered significant at *P *< 0.05. Bar graphs were analyzed using Graph Pad Prism (GraphPad, San Diego, CA, USA).

## Results

### Molecular docking evaluation

**Fig. 4:**
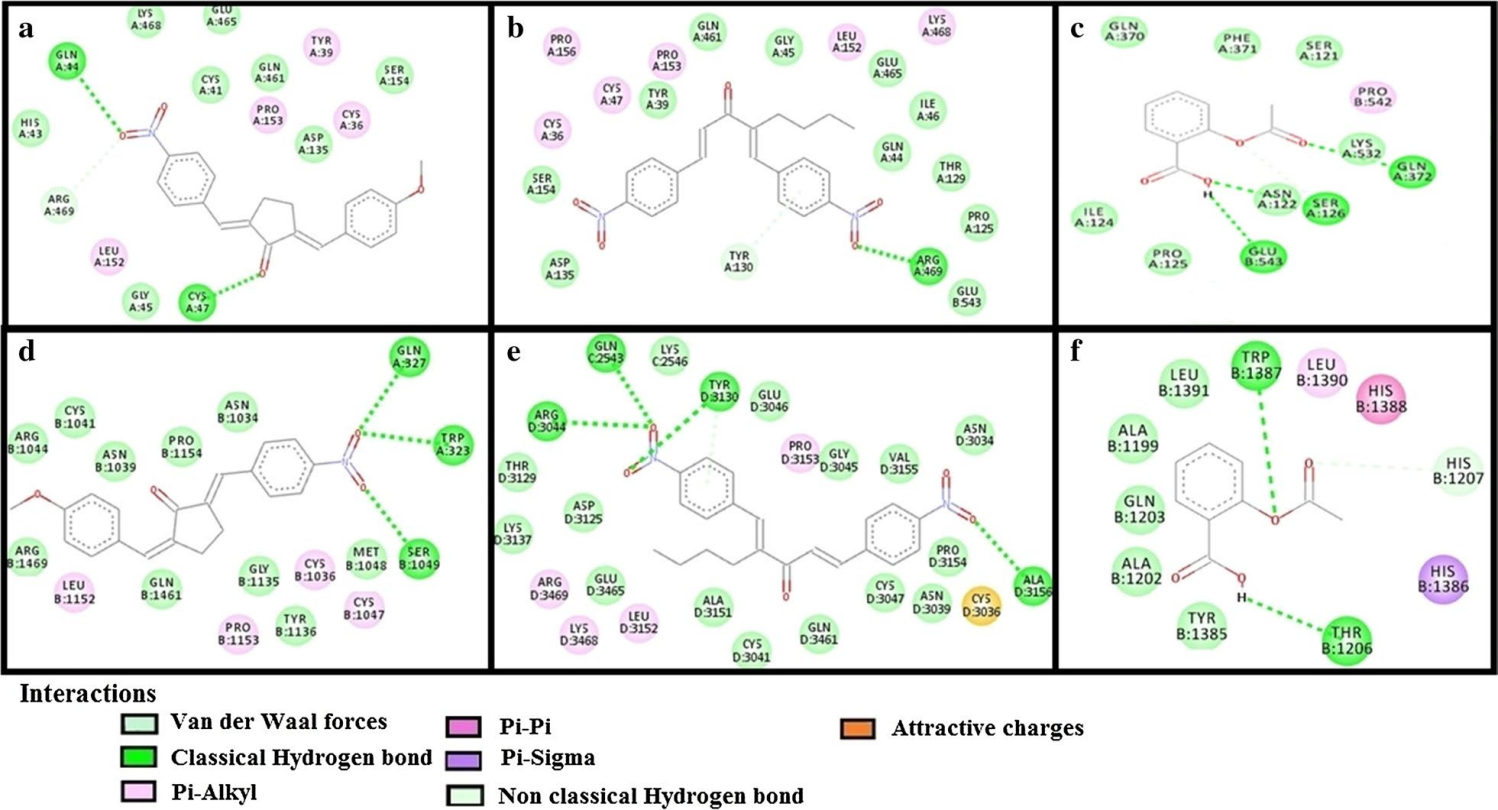
**a**–**c** Represents interactions of ligands: (2*E*,5*E*)-2-(4-methoxybenzylidene)-5-(4-nitrobenzylidene) cyclopentanone (AK-1a), (1*E*,4*E*)-4-(4-nitrobenzylidene)-1-(4-nitrophenyl)oct-1-en-3-one (AK-2a) and aspirin with target: cyclooxygenase-1 (COX-1) respectively. **d**–**f** Represents interactions of AK-1a, AK-2a and aspirin with target: cyclooxygenase-2 (COX-2) respectively, drawn through Biovia Discovery Studio Visualizer client 2016

The results of E-values, hydrogen bonds and binding residues of AK-1a and AK-2a with target proteins involved in pain pathways along with standard drugs are shown in Table 1 and Figs. 4 5, 6, 7. The results of E-values, hydrogen bonds and binding residues of AK-1a and AK-2a with target proteins involved in platelet aggregation along with standard drugs are shown in Table 2 and Figs. 7, 8, 9. The results of E-values, hydrogen bonds and binding residues of AK-1a and AK-2a with target proteins involved in coagulation process along with the standard drugs are shown in Table 3 and Figs. 10, 11, 12.

**Table 1:** E-value (kcal/mol) and post-docking analysis of best pose of (2E,5E)-2-(4-methoxybenzylidene)-5-(4-nitrobenzylidene) cyclopentanone (AK-1a), (1E,4E)-4-(4-nitrobenzylidene)-1-(4-nitrophenyl) oct-1-en-3-one (AK-2a) and standard drugs with cyclooxygenase-1 (COX-1), cyclooxygenase-2 (COX-2), mu receptor, kappa receptor, delta receptor, human capcaisin receptor (HCR) and purinoceptor-3 (P2X3)

Target proteins	PDB-IDs	AK-1a			AK-2a			Standard drugs			
		E-value (kcal/mol)	No. of H-bond	Binding residues	E-value (kcal/mol)	No. of H-bond	Binding residues	Standard	E-value (kcal/mol)	No. of H-bond	Binding residues
COX 1	3N8X	− 10.2	03	GLN-A:44 CYS-A:47 ARG-A:469	− 9.6	02	TYR-A:130 ARG-A:469	Aspirin	− 6.1	04	SER-A:126(2) GLN-A:372 GLU-B:543
COX 2	1PXX	− 9.7	03	TRP-A:323 GLN-A:327 SER-B:1049	− 9.6	05	GLN-C:2543 ARG-D:3044 TYR-D:3130(2) ALA-D:3156	Aspirin	− 7.6	03	THR-B:1206 HIS-B:1207 TRP-B:1387
Mu receptor	5C1M	− 8.7	02	ASN-A:127 HIS-A:297	-8.8	03	TYR-A:75 ASN-A:127 HIS-A:297	Morphine	− 8.5	02	HIS-A:297 ASP-A:147
Kappa receptor	4DJH	− 8.3	02	THR-A:63 TYR-B:313	− 8.4	03	THR-B:63(2) SER-B:116	Morphine	− 8.0	00	00
Delta receptor	4EJ4	− 8.2	03	VAL-A:75 LYS-A:166 ASN-A:169	− 8.0	02	LYS-A:108 HIS-A:278	Morphine	− 7.3	00	00
Human Capcaisin receptor	3J9J	− 8.8	02	ARG-C:177 GLY-C:183	− 8.9	01	ARG-D:177	Capsazepine	− 8.2	03	ASN-A:57 SER-A:103 TYR-A:107
P2X3	5SVL	− 7.4	01	TRP-A:41	− 6.9	02	LYS-A:65 PHE-A:205	Capsazepine	− 5.4	02	ASP-A:266 ASN-A:279

*GLN* glutamine, *CYS* cysteine, *ARG* arginine, *TYR* tyrosine, *SER* serine, *GLU* glutamic acid, *TRP* tryptophan, *ALA* alanine, *THR* threonine, *HIS* histidine, *ASN* asparagine, *VAL* valine, *LYS* lysine, *GLY* glycine, *PHE* phenylalanine, *ASP* aspartic acid

**Fig. 5:**
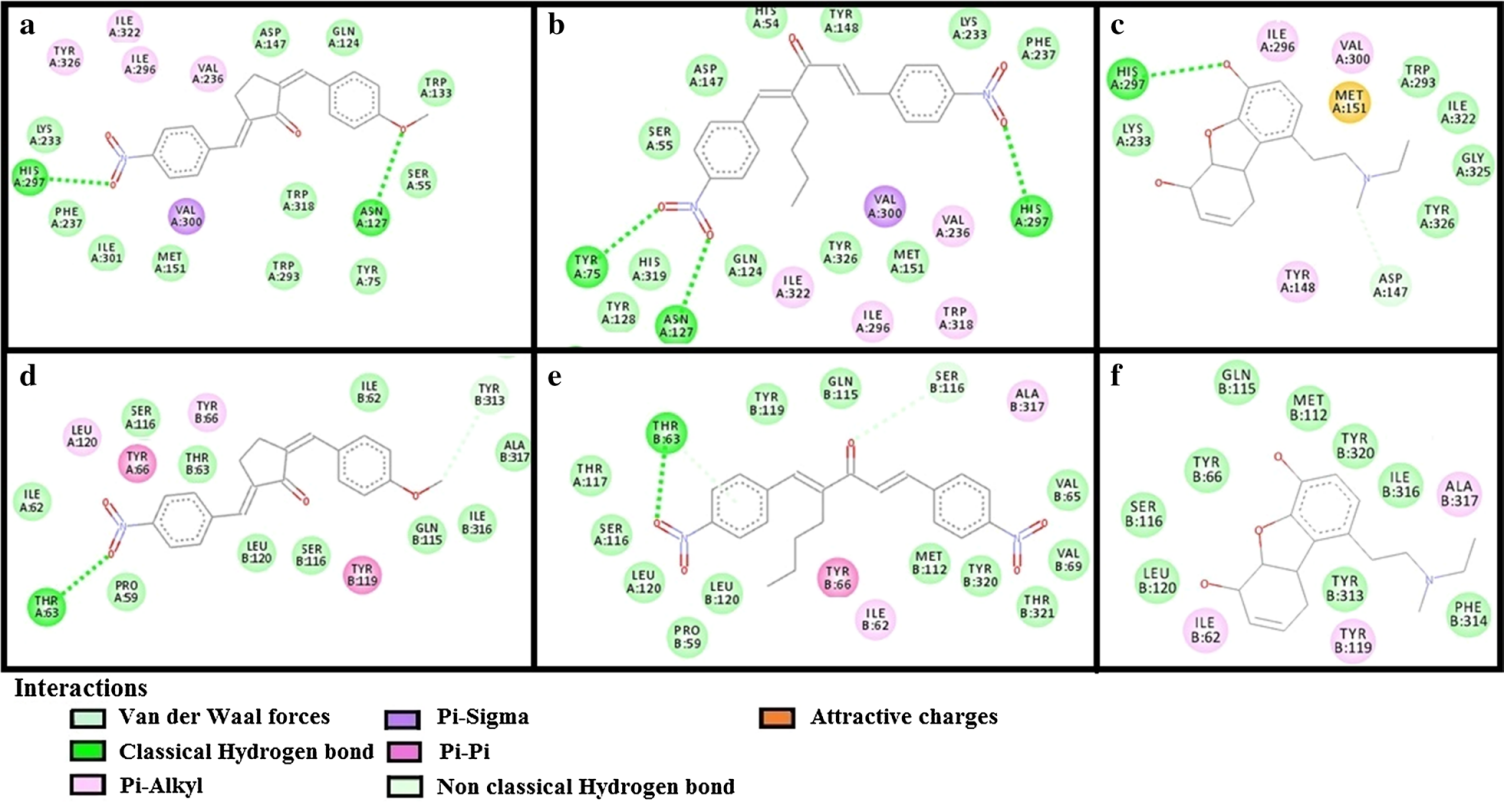
**a**–**c** Represents interactions of ligands: (2*E*,5*E*)-2-(4-methoxybenzylidene)-5-(4-nitrobenzylidene) cyclopentanone (AK-1a), (1*E*,4*E*)-4-(4-nitrobenzylidene)-1-(4-nitrophenyl) oct-1-en-3-one (AK-2a) and morphine with target: mu receptor respectively. **d**–**f** Represents interactions of AK-1a, AK-2a and morphine with target: kappa receptor respectively, drawn through Biovia Discovery Studio Visualizer client 2016

**Fig. 6:**
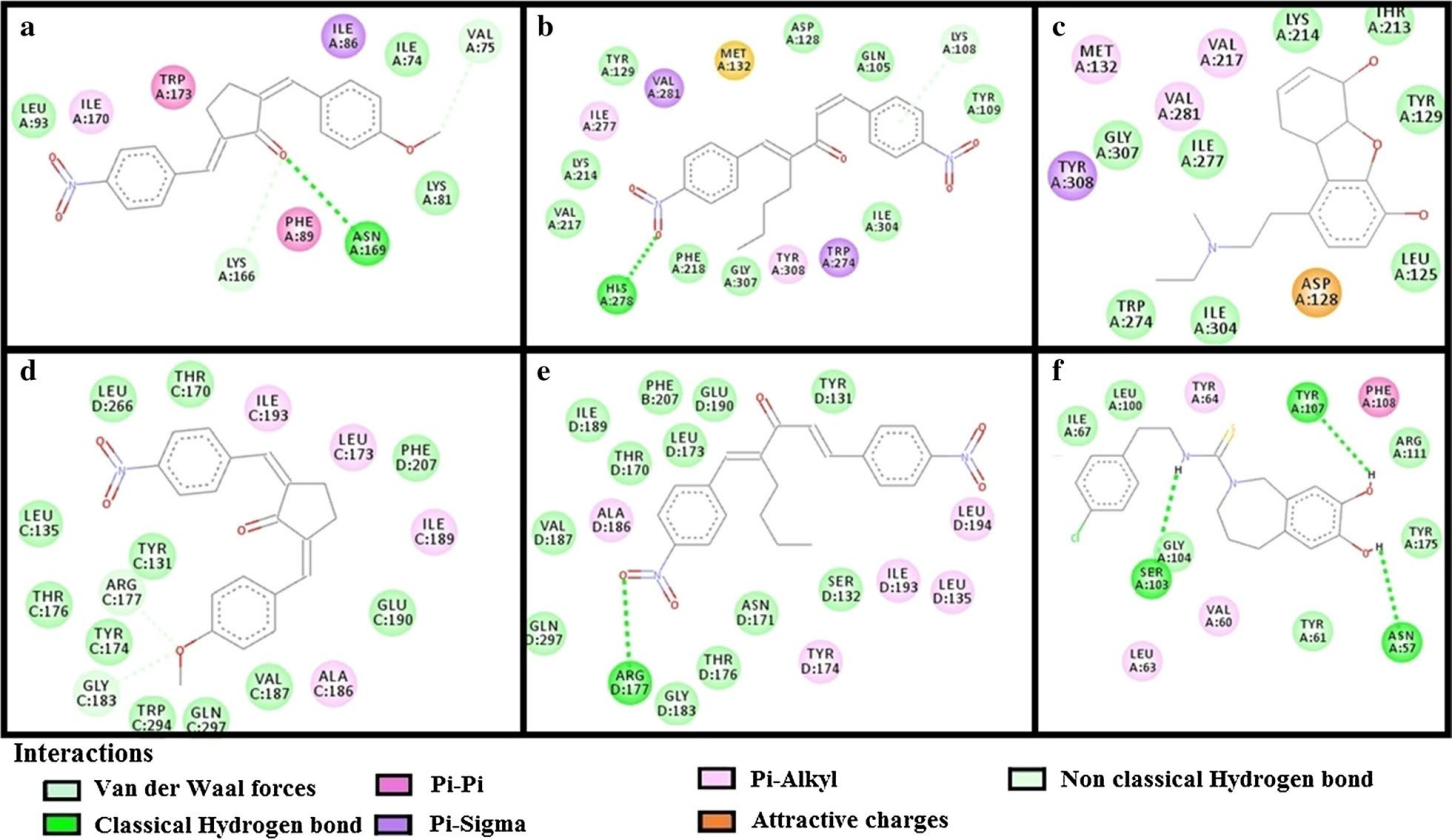
**a**–**c** Represents interactions of ligands: (2*E*,5*E*)-2-(4-methoxybenzylidene)-5-(4-nitrobenzylidene) cyclopentanone (AK-1a), (1*E*,4*E*)-4-(4-nitrobenzylidene)-1-(4-nitrophenyl) oct-1-en-3-one (AK-2a) and morphine with target: delta receptor respectively. **d**–**f** Represents interactions of AK-1a, AK-2a and capsazepine with target: human capsaicin receptor (HCR) respectively, drawn through Biovia Discovery Studio Visualizer client 2016

**Fig. 7:**
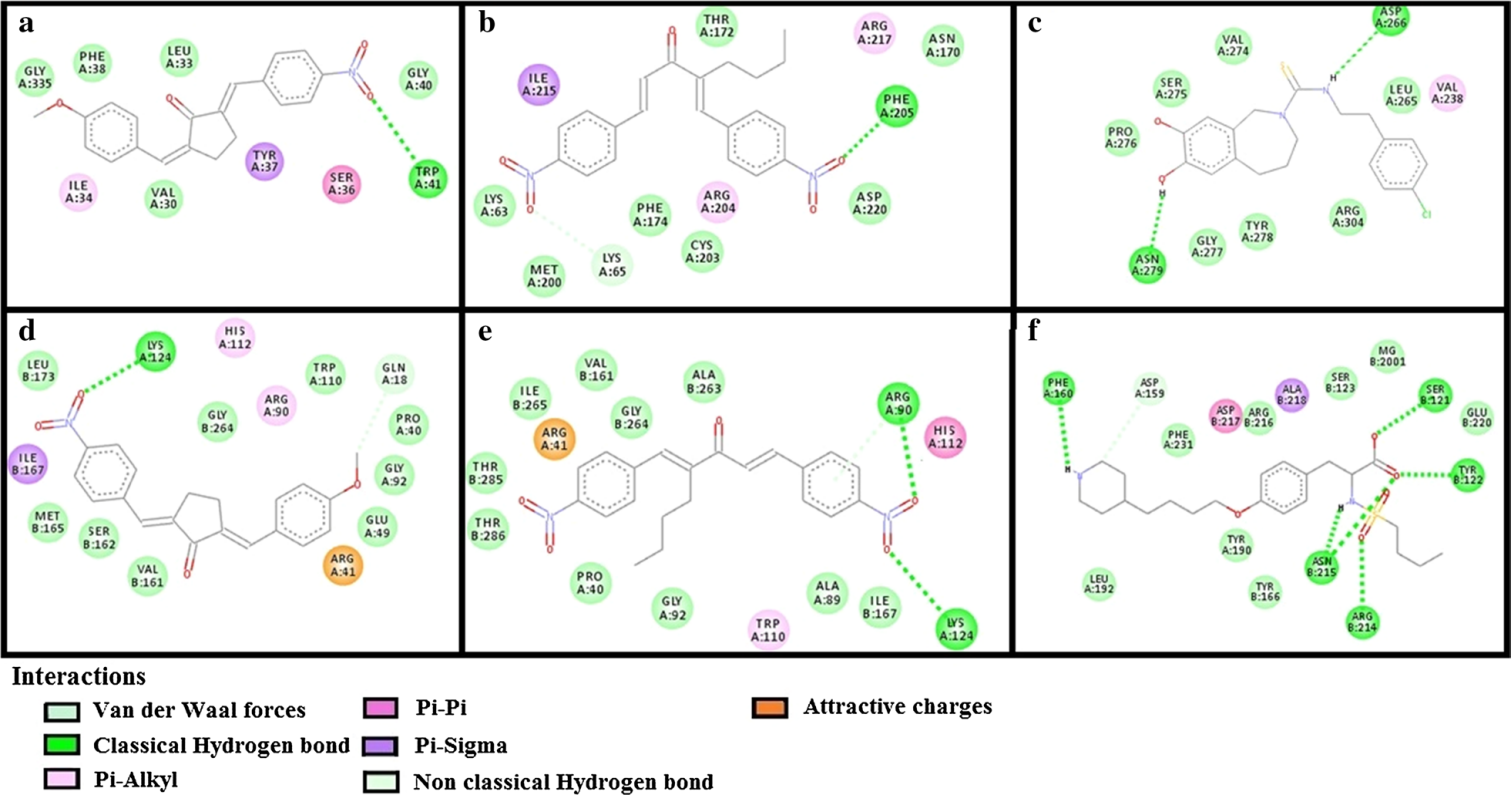
**a**–**c** Represents interactions of ligands: (2*E*,5*E*)-2-(4-methoxybenzylidene)-5-(4-nitrobenzylidene) cyclopentanone (AK-1a), (1*E*,4*E*)-4-(4-nitrobenzylidene)-1-(4-nitrophenyl) oct-1-en-3-one (AK-2a) and capsazepine with target: purinoceptor-3 (P2X3) respectively. **d**–**f** Represents interactions of AK-1a, AK-2a and tirofiban with target: glycoprotein-IIb/IIIa (GP-IIb/IIIa) respectively, drawn through Biovia Discovery Studio Visualizer client 2016

**Table 2:** E-value (Kcal/mol) and post-docking analysis of best pose of (2E,5E)-2-(4-methoxybenzylidene)-5-(4-nitrobenzylidene) cyclopentanone (AK-1a), (1E,4E)-4-(4-nitrobenzylidene)-1-(4-nitrophenyl) oct-1-en-3-one (AK-2a) and standard drugs with cyclooxygenase-1 (C0X-1), glycoprotein IIb/IIIa (GP- IIb/IIIa), glycoprotein-VI (GP-VI), purinergic receptor P_2_Y_12_ (P_2_Y_12_), prostacyclin receptor I_2_ (PG-I_2_) and proteinase-activated receptor 1 (PAR-1)

Target proteins	PDB-IDs	AK-1a			AK-2a			Standard drugs			
		E-value (kcal/mol)	No. of H-bond	Bonding residues	E-value (kcal/mol)	No. of H-bond	Bonding residues	Standard	E-value (kcal/mol)	No. of H-bond	Bonding residues
COX-1	3N8X	− 10.2	03	GLN-A:44 CYS-A:47 ARG-A:469	− 9.6	02	TYR-A:130 ARG-A:469	Aspirin	− 6.1	04	SER-A:126(2) GLN-A:372 GLU-B:543
GP-IIb/IIIa	2VdM	− 8.7	02	GLN-A:18 LYS-A:124	− 8.4	03	ARG-A:90(2) LYS-A:124	Tirofiban	− 7.9	07	SER-B:121 TYR-B:122 ASP-A:159 PHE-A:160 ARG-B:214 ASN-B:215(2)
GP-VI	2G17	− 7.1	02	SER-A:69(2)	− 6.8	02	TYR-A:126,161	Hinokitiol	− 5.8	01	SER-A:16
P_2_Y_12_	4PXZ	− 8.5	02	LYS-A:64 ASN-A:65	− 7.3	00	00	Clopidogrel	− 6.0	04	SER-A:113(2) ASN-A:201(2)
PG-I_2_	4F8K	− 8.9	03	ILE-A:05 GLU-A:09 UNK-A:12	− 8.4	02	GLU-A:09 UNK-A:12	Beraprost	− 8.3	02	ARG-B:36 LEU-B:74
PAR-1	3VW7	− 10.4	02	TYR-A:187 GLY-A:233	− 10.4	02	TYR-A:187,337	Vorapaxar	− 12.4	06	ASP-A:256 VAL-A:257 LEU-A:258 TYR-A:337 ALA-A:349(2)

*GLN* glutamine, *CYS* cysteine, *ARG* arginine, *TYR* tyrosine, *SER* serine, *GLU* glutamic acid, *TRP* tryptophan, *ALA* alanine, *THR* threonine, *HIS* histidine, *ASN* asparagine, *VAL* valine, *LYS* lysine, *LEU* leucine, *ILE* isoleucine, *GLY* glycine, *PHE* phenylalanine, *ASP* aspartic acid

**Fig. 8:**
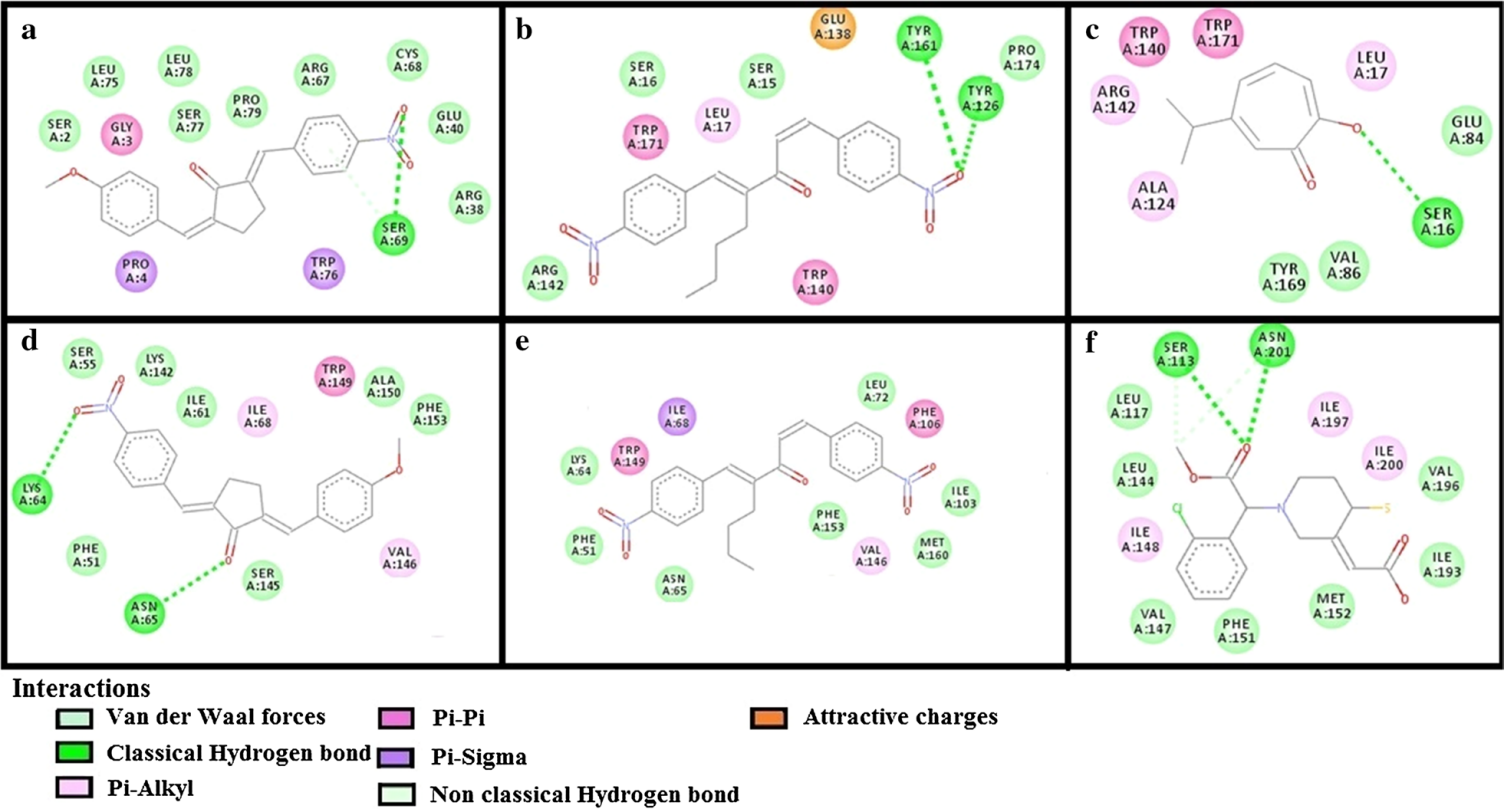
**a**–**c** Represents interactions of ligands: (2*E*,5*E*)-2-(4-methoxybenzylidene)-5-(4-nitrobenzylidene) cyclopentanone (AK-1a), (1*E*,4*E*)-4-(4-nitrobenzylidene)-1-(4-nitrophenyl) oct-1-en-3-one (AK-2a) and hinokitiol with target: glycoprotein-VI (GP-VI) respectively. **d**–**f** represents interactions of AK-1a, AK-2a and clopidogrel with target: purinergic receptor (P_2_Y_12_) respectively, drawn through Biovia Discovery Studio Visualizer client 2016

**Fig. 9:**
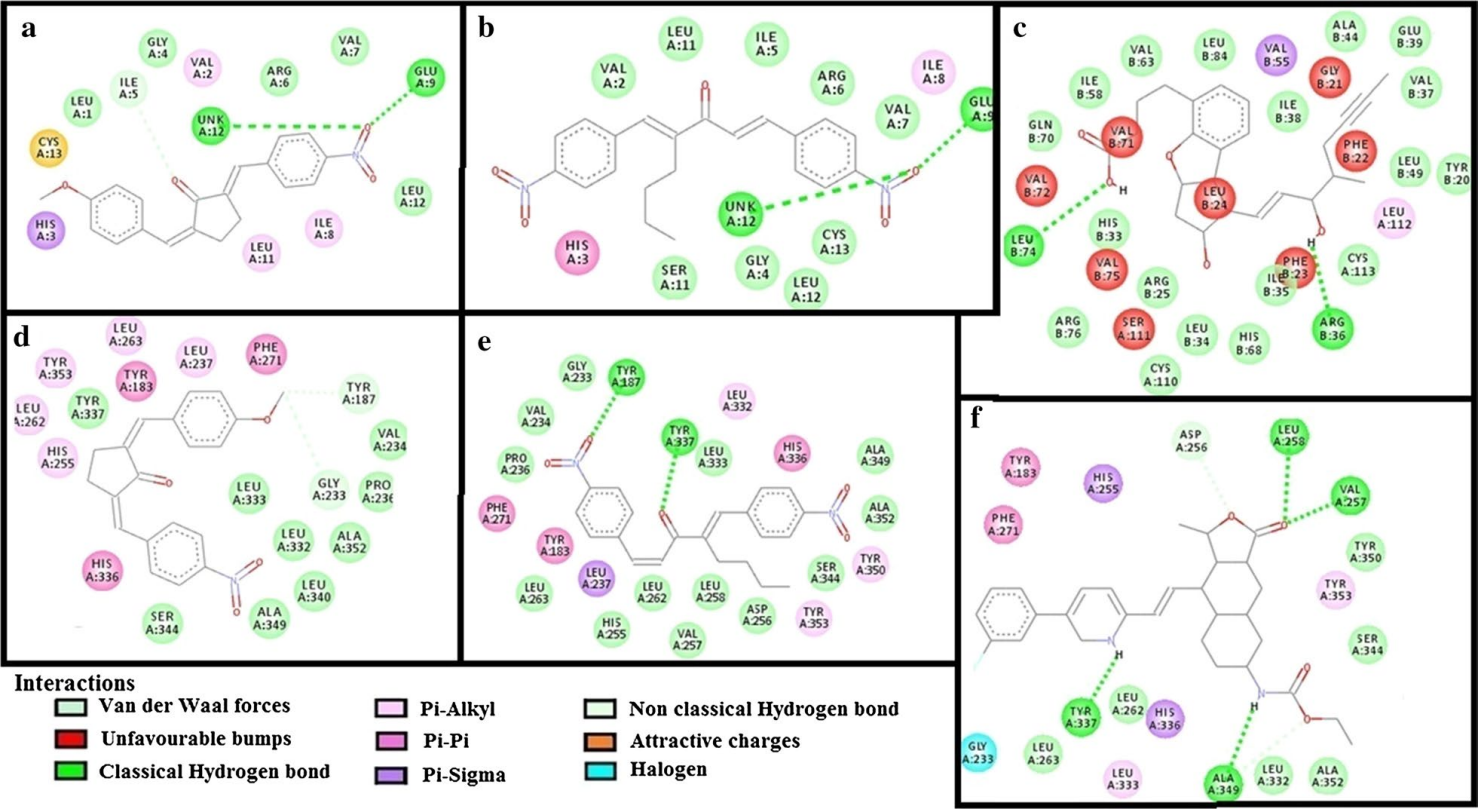
**a**–**c** Represents interactions of ligands: (2*E*,5*E*)-2-(4-methoxybenzylidene)-5-(4-nitrobenzylidene)cyclopentanone (AK-1a), (1*E*,4*E*)-4-(4-nitrobenzylidene)-1-(4-nitrophenyl) oct-1-en-3-one (AK-2a) and beraprost with target: prostacyclin receptor I_2_ (PG-I_2_) respectively. **d**–**f** Represents interactions of AK-1a, AK-2a and vorapaxar with target: proteinase-activated receptor 1 (PAR-1) respectively, drawn through Biovia Discovery Studio Visualizer client 2016

**Table 3:** E-value (kcal/mol) and post-docking analysis of best pose of (2E,5E)-2-(4-methoxybenzylidene)-5-(4-nitrobenzylidene) cyclopentanone (AK-1a), (1E,4E)-4-(4-nitrobenzylidene)-1-(4-nitrophenyl) oct-1-en-3-one (AK-2a) and standard drugs with antithrombin-III (AT-III), factor-X (F-X), factor-II (F-II), factor-IX (F-IX) and vitamin-K epoxide reductase (VKOR)

Target proteins	PDB-IDs	AK-1a			AK-2a			Standard drugs			
		E-value (kcal/mol)	No. of H-bond	Bonding residues	E-value (kcal/mol)	No. ofH-bond	Bonding residues	Standard	E-value (kcal/mol)	No. of H-bond	Bonding residues
AT-III	2B4X	− 8.1	00	00	− 8.2	02	ASN-I:233 ARG-I:399	Heparin SO_4_	− 4.1	06	ASN-I:233 GLN-L:268(2) VAL-I:388 ARG-I:393(2)
F-II	1XMN	− 8.7	01	GLY-B:223	− 8.1	03	LYS-D:169 GLY-D:223 TYR-D:225	Argatroban	− 8.0	08	GLU-D:39 LEU-D:40 LEU-D:41 ASN-D:143 THR-D:147B ALA-D:147C GLU-D:192
F-IX	1RFN	− 9.1	02	ASN-A:48 GLY-B:114	− 8.2	04	ASN-A:97 THR-A:175 SER-A:195 GLN-A:192	Pegnivacogin	− 7.6	00	3D image not found
F-X	1KSN	− 8.3	01	GLN-A:192	− 8.2	03	TYR-A:99 SER-A:195 GLU-A:217	Apixaban	− 9.2	03	TYR-A:99 GLN-A:192 SER-A:195
VKOR	3KP9	− 10.3	03	ALA-A:110 MET-A:111,122	− 7.6	00	00	Warfarin	− 12.4	02	THR-A:34 LYS-A:41

*GLN* glutamine, *CYS* cysteine, *ARG* arginine, *TYR* tyrosine, *SER* serine, *MET* methionine, *GLU* glutamic acid, *TRP* tryptophan, *ALA* alanine, *THR* threonine, *HIS* histidine, *ASN* asparagine, *VAL* valine, *LYS* lysine, *LEU* leucine, *ILE* isoleucine, *GLY* glycine, *PHE* phenylalanine, *ASP* aspartic acid

**Fig. 10:**
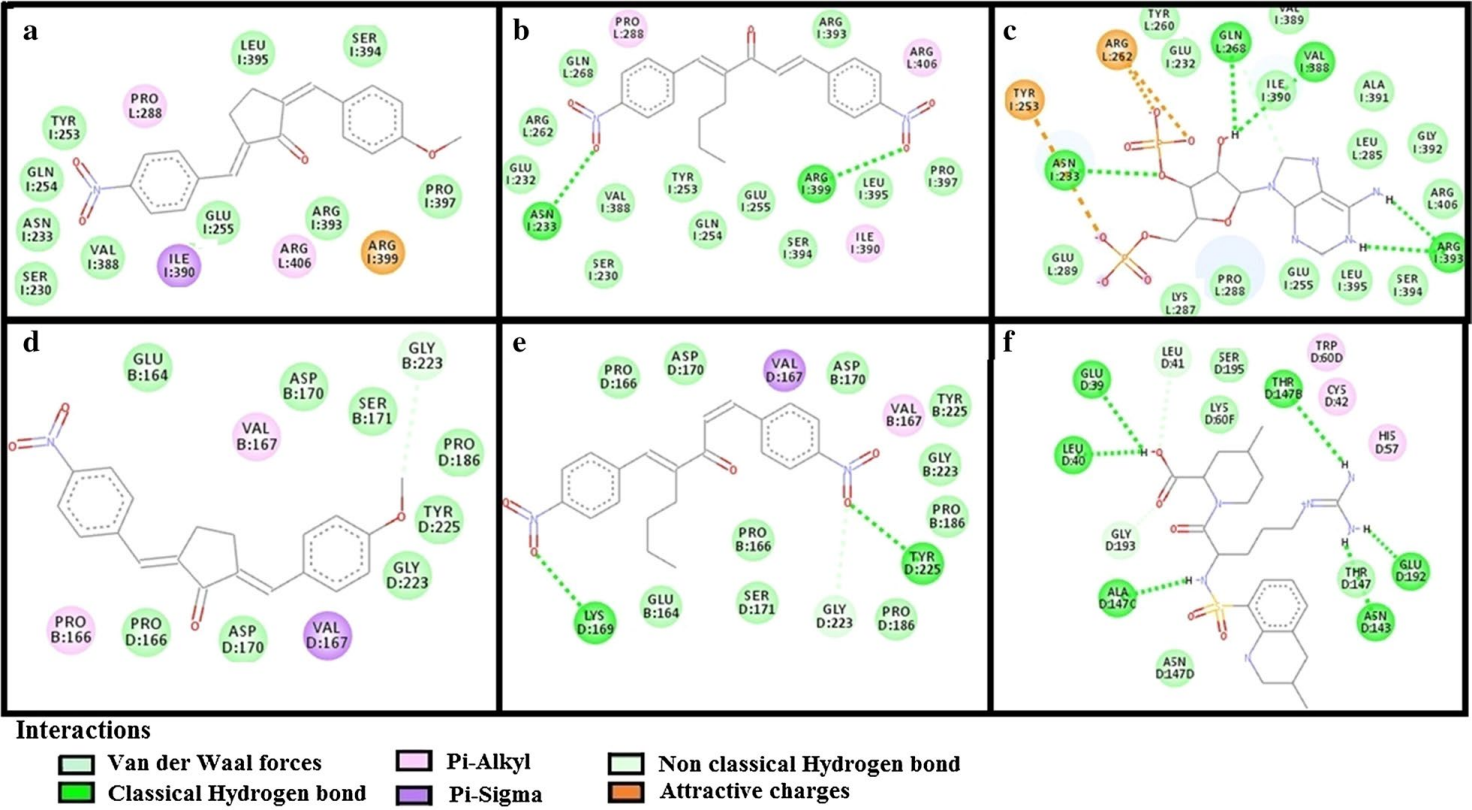
**a**–**c** Represents interactions of ligands: (2*E*,5*E*)-2-(4-methoxybenzylidene)-5-(4-nitrobenzylidene)cyclopentanone (AK-1a), (1*E*,4*E*)-4-(4-nitrobenzylidene)-1-(4-nitrophenyl) oct-1-en-3-one (AK-2a) and heparin sulphate with target: antithrombin-III (AT-III) respectively. **d**–**f** Represents interactions of AK-1a, AK-2a and argatroban with target: factor-II (F-II) respectively, drawn through Biovia Discovery Studio Visualizer client 2016

**Fig. 11:**
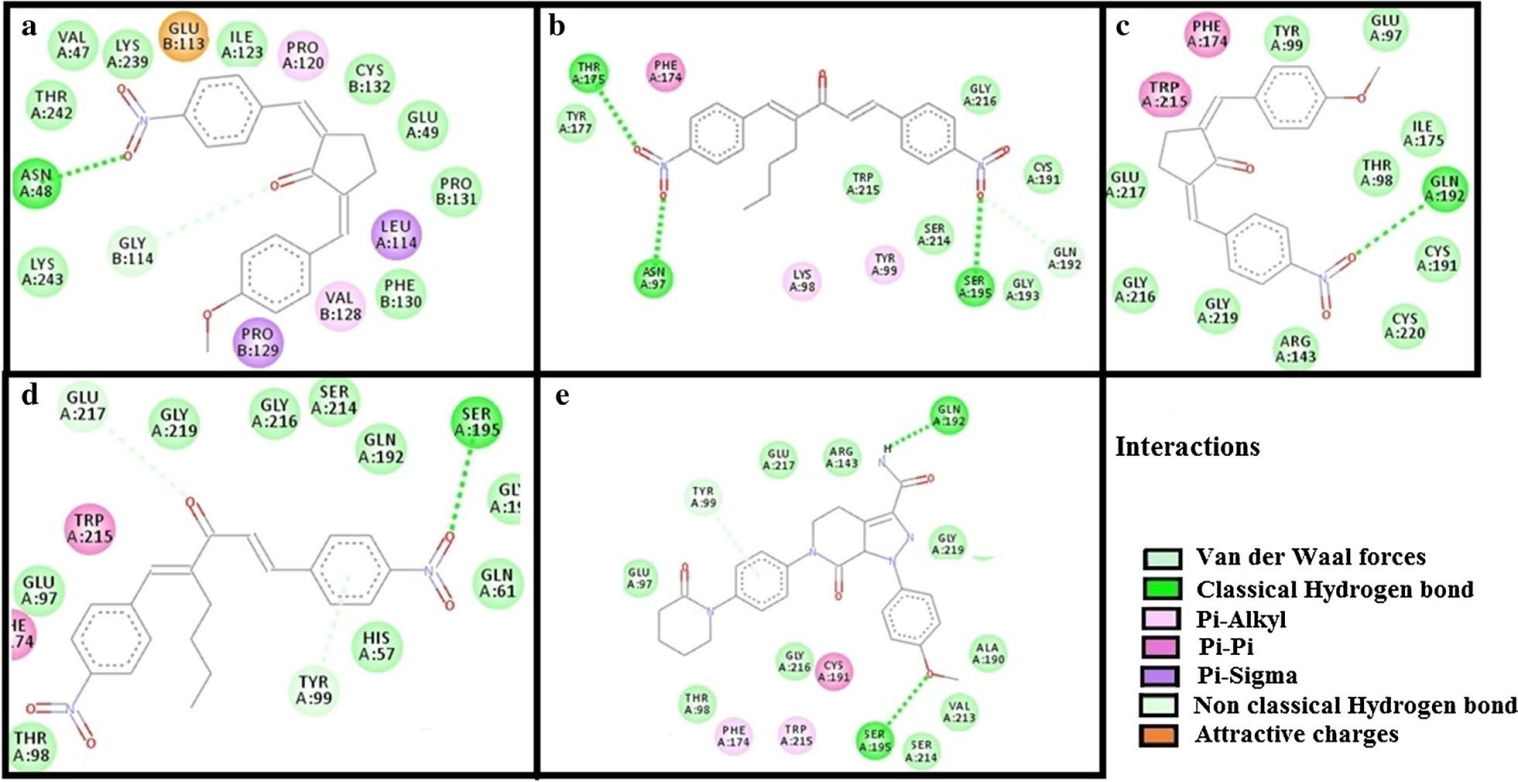
**a**, **b** Represents interactions of ligands: (2*E*,5*E*)-2-(4-methoxybenzylidene)-5-(4-nitrobenzylidene) cyclopentanone (AK-1a) and (1*E*,4*E*)-4-(4-nitrobenzylidene)-1-(4-nitrophenyl) oct-1-en-3-one (AK-2a) with target factor-IX (F-IX) respectively. **c**–**e** Represents interaction of AK-1a, AK-2a and apixaban with target: factor-X (F-X) respectively, drawn through Biovia Discovery Studio Visualizer client 2016

**Fig. 12:**
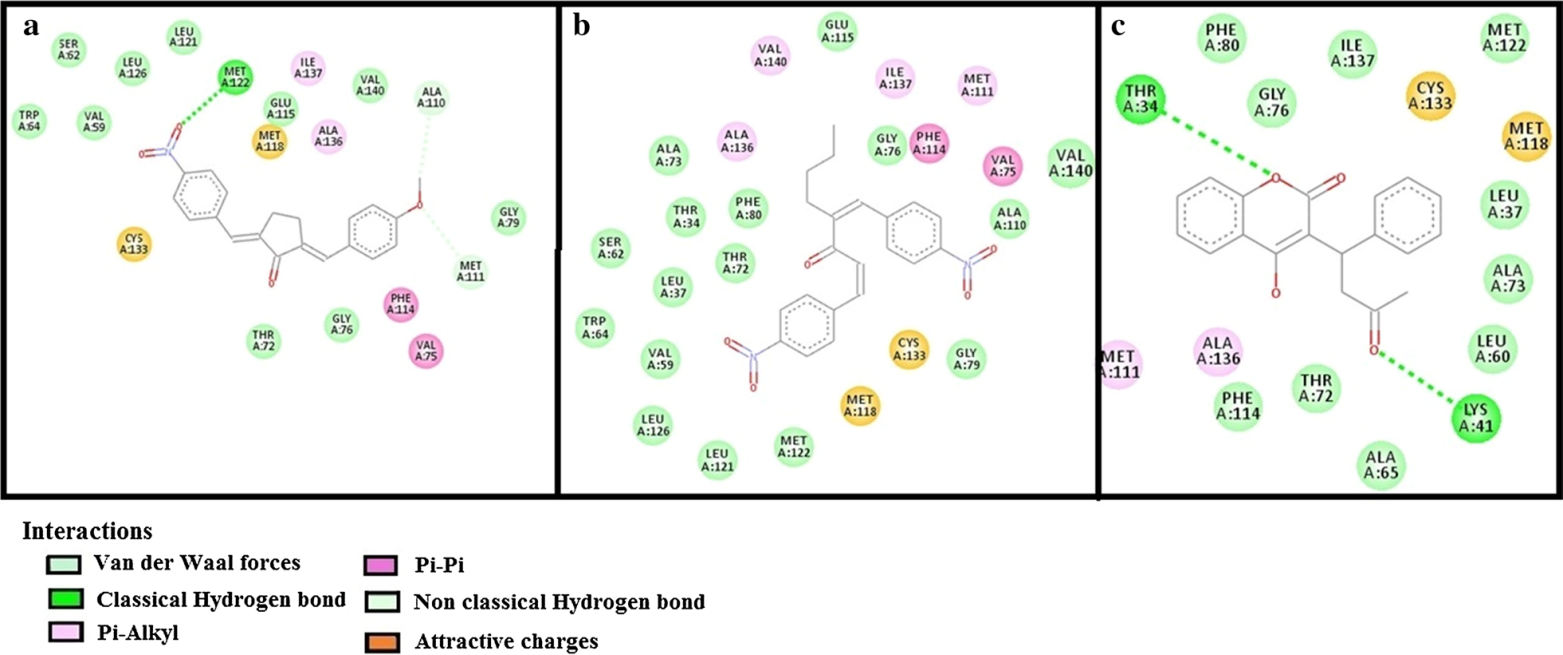
**a**–**c** Represents interactions of ligands: (2*E*,5*E*)-2-(4-methoxybenzylidene)-5-(4-nitrobenzylidene) cyclopentanone (AK-1a), (1*E*,4*E*)-4-(4-nitrobenzylidene)-1-(4-nitrophenyl)oct-1-en-3-one (AK-2a) and warfarin with target: vitamin-K epoxide reductase (VKOR) respectively, drawn through Biovia Discovery Studio Visualizer client 2016

### Effect on acetic acid-induced writhings

**Fig. 13:**
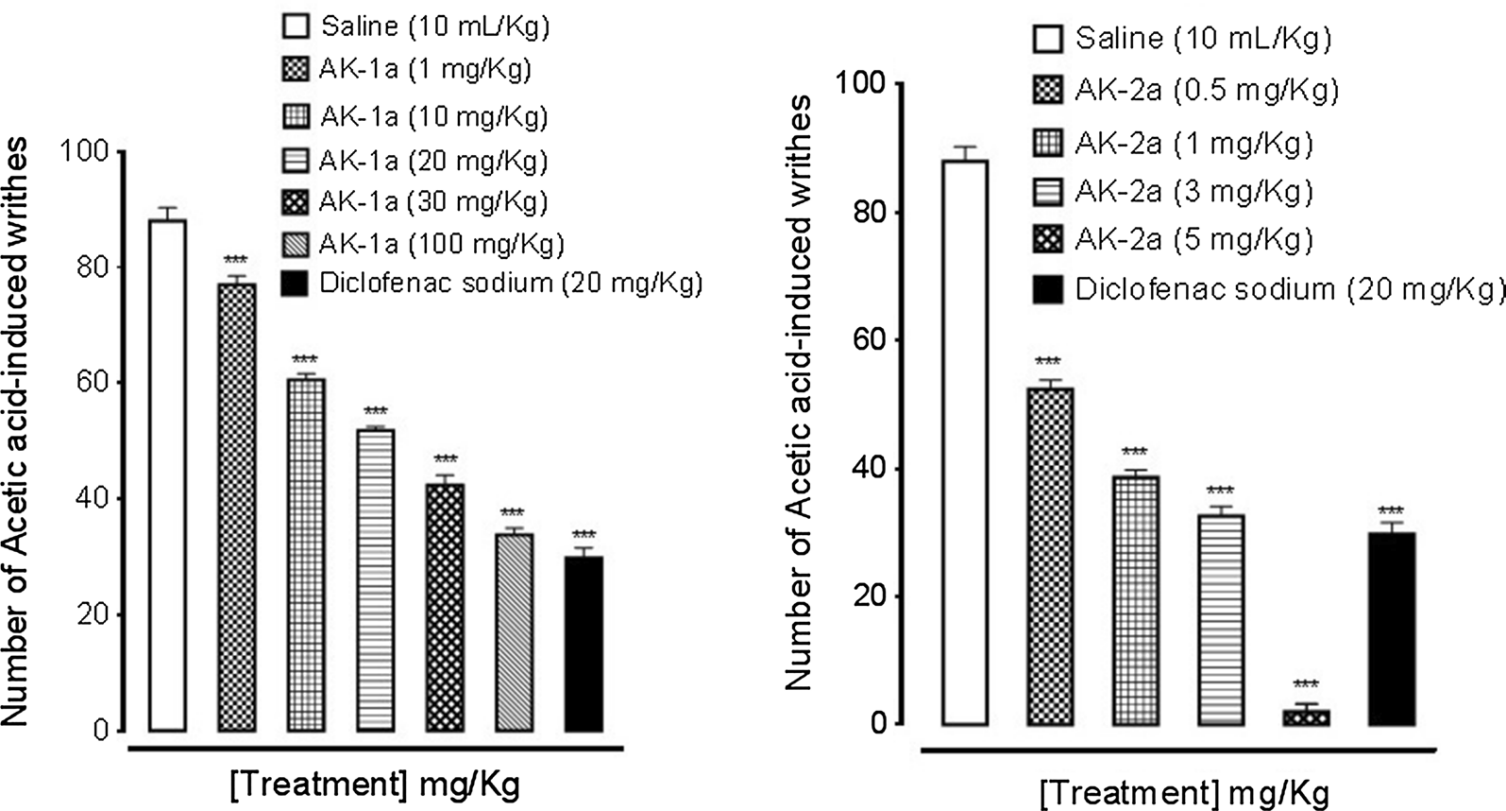
Effect of (2*E*,5*E*)-2-(4-methoxybenzylidene)-5-(4-nitrobenzylidene) cyclopentanone (AK-1a), (1*E*,4*E*)-4-(4-nitrobenzylidene)-1-(4-nitrophenyl) oct-1-en-3-one (AK-2a) and diclofenac sodium on acetic acid-induced writhes in mice. Data expressed as mean ± SEM, n = 5. ***P < 0.001 vs. saline group, one way ANOVA with post hoc Tukey’s test

Saline group (10 mL/kg) showed 88 ± 2.28 numbers of writhes. The writhes count in AK-1a treated group (1, 10, 20, 30 and 100 mg/kg) were decreased to 77 ± 1.51, 60.60 ± 1.07, 51.80 ± 0.73, 42.40 ± 1.72 and 33.80 ± 1.20 (*P *< 0.001 vs. saline group) respectively. Diclofenac sodium (20 mg/kg) decreased numbers of writhes to 29.80 ± 1.77 (*P *< 0.001 vs. saline group). AK-2a showed significant response in acetic acid induced writhing. The writhes count in AK-2a treated group (0.5, 1, 3 and 5 mg/kg) were decreased to 52.40 ± 1.40, 38.60 ± 1.20, 32.60 ± 1.50 and 2.00 ± 1.26 (*P *< 0.001 vs. saline group) respectively as shown in Fig. 13.

### Effect on latency time

**Fig. 14:**
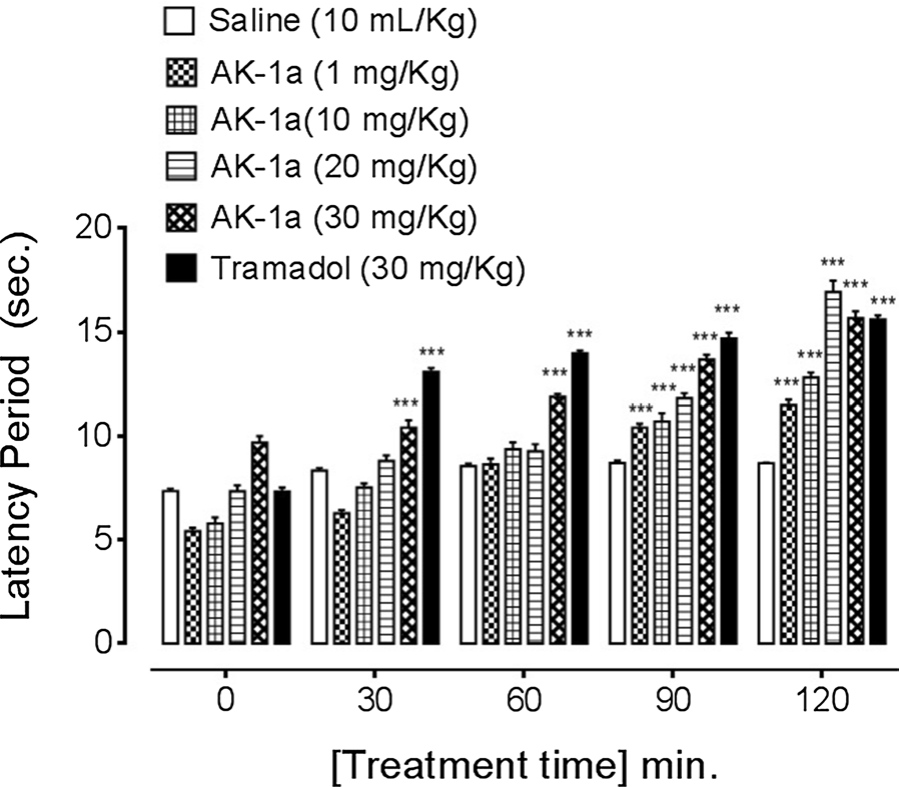
Effect of (2*E*,5*E*)-2-(4-methoxybenzylidene)-5-(4-nitrobenzylidene) cyclopentanone (AK-1a) and tramadol on latency time in hot plate assay. Data expressed as mean ± SEM, n = 5. ***P < 0.001 vs. saline group, one way ANOVA with post hoc Tukey’s test

**Fig. 15:**
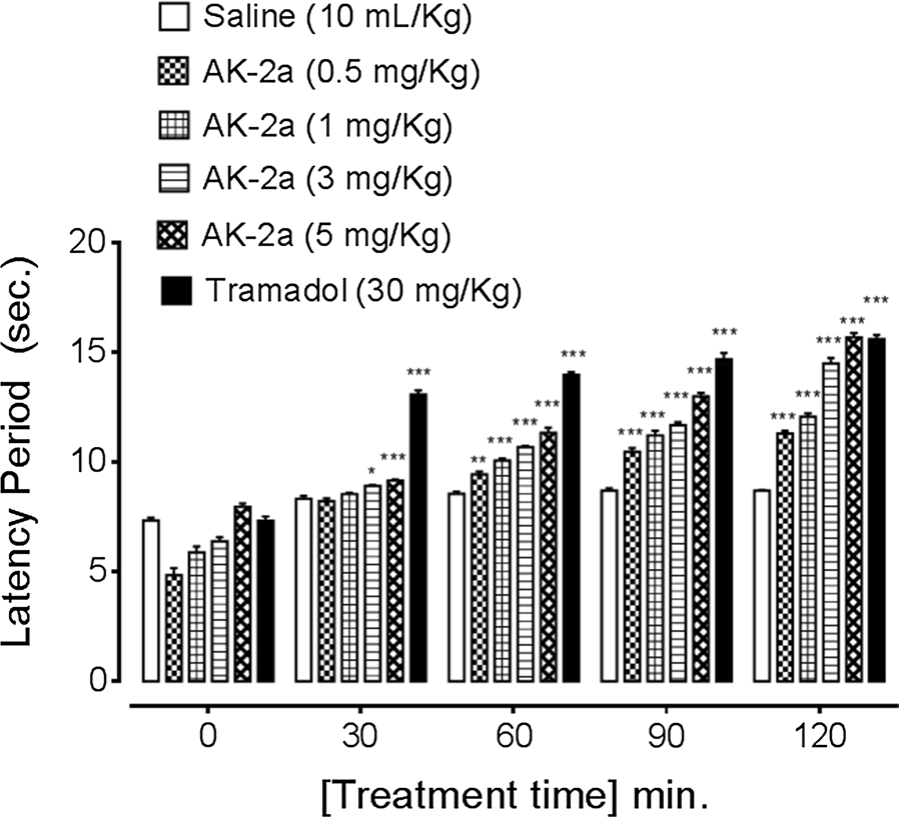
Effect of (1*E*,4*E*)-4-(4-nitrobenzylidene)-1-(4-nitrophenyl) oct-1-en-3-one (AK-2a) and tramadol on latency time in hot plate assay. Data expressed as mean ± SEM, n = 5. *P < 0.05, **P < 0.01 and ***P < 0.001 vs. saline group, one way ANOVA with post hoc Tukey’s test

The latency time of saline group (10 mL/kg) at 0, 30, 60, 90 and 120 min were 7.35 ± 0.12, 8.33 ± 0.13, 8.56 ± 0.10, 8.71 ± 0.10 and 8.70 ± 0.03 s respectively. AK-1a dose dependently (1, 10, 20 and 30 mg/kg) prolonged latency time against thermal pain generation. The latency time of AK-1a (1 mg/kg) treated group at 0, 30, 60, 90 and 120 min were 5.42 ± 0.15, 6.28 ± 0.15, 8.63 ± 0.28, 10.40 ± 0.19, 11.47 ± 0.27 s (*P *< 0.001 vs. saline group) respectively. The latency time of AK-1a (10 mg/kg) treated group at 0, 30, 60, 90, 120 min were 5.78 ± 0.28, 7.51 ± 0.20, 9.36 ± 0.32, 10.71 ± 0.39 and 12.80 ± 0.24 s (*P *< 0.001 vs. saline group) respectively. The latency time of AK-1a (20 mg/kg) treated group at 0, 30, 60, 90 and 120 min were 7.33 ± 0.29, 8.81 ± 0.26, 9.27 ± 0.33, 11.81 ± 0.24 and 16.92 ± 0.55 s (*P *< 0.001 vs. saline group) respectively. The latency time of AK-1a (30 mg/kg) treated group at 0, 30, 60, 90 and 120 min were 9.69 ± 0.31, 10.40 ± 0.36, 11.88 ± 0.13, 13.67 ± 0.23 and 15.66 ± 0.33 s (*P *< 0.001 vs. saline group) respectively. The latency time of tramadol (30 mg/kg) treated group at 0, 30, 60, 90 and 120 min were 7.33 ± 0.20, 13.07 ± 0.18, 13.97 ± 0.12, 14.69 ± 0.27 and 15.61 ± 0.18 s (*P *< 0.001 vs. saline group) respectively as shown in Fig. 14. AK-2a dose dependently (0.5, 1, 3 and 5 mg/kg) prolonged latency time against thermal pain generation. The latency time of AK-2a (0.5 mg/kg) treated group at 0, 30, 60, 90 and 120 min were 4.85 ± 0.32, 8.23 ± 0.12, 9.45 ± 0.12 s (*P *< 0.01 vs. saline group), 10.48 ± 0.17 and 11.32 ± 0.12 s (*P *< 0.001 vs. saline group) respectively. The latency time of AK-2a (1 mg/kg) treated group at 0, 30, 60, 90 and 120 min were 5.89 ± 0.26, 8.55 ± 0.06, 10.080 ± 0.105, 11.23 ± 0.21 and 12.06 ± 0.15 s (*P *< 0.001 vs. saline group) respectively. The latency time of AK-2a (3 mg/kg) treated group at 0, 30, 60, 90 and 120 min were 6.39 ± 0.18, 8.93 ± 0.03 (*P *< 0.05 vs. saline group), 10.70 ± 0.05, 11.70 ± 0.12 and 14.49 ± 0.25 s (*P *< 0.001 vs. saline group) respectively. The latency time of AK-2a (5 mg/kg) treated group at 0, 30, 60, 90 and 120 min were 7.96 ± 0.15, 9.16 ± 0.04, 11.36 ± 0.23, 12.99 ± 0.15 and 15.69 ± 0.19 s (*P *< 0.001 vs. saline group) respectively as shown in Fig. 15.

### Effect on AA-induced platelet aggregation inhibition

AK-1a at 1, 3, 10, 30, 100, 300 and 1000 µM concentrations, inhibited AA-induced platelet aggregation to 2.3 ± 0.06, 7.2 ± 0.06, 20.4 ± 0.06, 33.2 ± 0.14, 55.6 ± 0.20, 67.1 ± 0.15 and 88.5 ± 0.18% respectively with IC_50_ value of 65.2 µM. At same concentrations AK-2a inhibited AA-induced platelet aggregation to 4.3 ± 0.07, 10.5 ± 0.09, 28 ± 0.15, 42.7 ± 0.22, 62.2 ± 0.08, 78.9 ± 0.19 and 89.8 ± 0.13% respectively with IC_50_ value of 37.7 µM. Aspirin inhibited AA-induced platelet aggregation to 27.2 ± 0.18, 36 ± 0.09, 50.1 ± 0.16, 59.7 ± 0.09 and 100% respectively with IC_50_ value of 10.01 µM, as shown in Table 4.

**Table 4:** Inhibitory effect of (2E,5E)-2-(4-methoxybenzylidene)-5-(4-nitrobenzylidene) cyclopentanone (AK-1a) and (1E,4E)-4-(4-nitrobenzylidene)-1-(4-nitrophenyl)oct-1-en-3-one (AK-2a) and aspirin against arachidonic acid (AA) and adenosine diphosphate (ADP)-induced platelet aggregation

Treatment	Agonist	Inhibition of platelet aggregation (%) ± SEM							IC_50_ (µM)
		1 µM	3 µM	10 µM	30 µM	100 µM	300 µM	1000 µM	
AK-1a	AA	2.3 ± 0.06	7.2 ± 0.06	20.4 ± 0.06	33.2 ± 0.14	55.6 ± 0.20	67.1 ± 0.15	88.5 ± 0.18	65.2
	ADP	1.81 ± 0.04	4.4 ± 0.04	13.4 ± 0.06	22.4 ± 0.04	31 ± 0.06	42.6 ± 0.06	54.1 ± 0.06	750.4
AK-2a	AA	4.3 ± 0.07	10.5 ± 0.09	28 ± 0.15	42.7 ± 0.22	62.2 ± 0.08	78.9 ± 0.19	89.8 ± 0.13	37.7
	ADP	4.4 ± 0.04	4.4 ± 0.07	14.2 ± 0.02	18.6 ± 0.06	30.2 ± 0.07	48.3 ± 0.12	56.8 ± 0.06	422
Aspirin	AA	27.2 ± 0.18	36 ± 0.09	50.1 ± 0.16	59.7 ± 0.09	100 ± 0	100 ± 0	100 ± 0	10.01
	ADP	3.6 ± 0.07	6.2 ± 0.09	19.1 ± 0.07	25 ± 0.06	32.8 ± 0.10	49.8 ± 0.12	56.9 ± 0.18	308.4

Values shown as mean ± SEM, n = 4

### Effect on ADP-induced platelet aggregation inhibition

AK-1a at 1, 3, 10, 30, 100, 300 and 1000 µM concentrations, inhibited ADP-induced platelet aggregation to 1.81 ± 0.04, 4.4 ± 0.04, 13.4 ± 0.06, 22.4 ± 0.04, 31 ± 0.06, 42.6 ± 0.06 and 54.1 ± 0.06% respectively with IC_50_ value of 750.4 µM. At same concentrations AK-2a inhibited ADP-induced platelet aggregation to 4.4 ± 0.04, 4.4 ± 0.07, 14.2 ± 0.02, 18.6 ± 0.06, 30.2 ± 0.07, 48.3 ± 0.12 and 56.8 ± 0.06% respectively with IC_50_ value of 422 µM. Aspirin inhibited ADP-induced platelet aggregation to 3.6 ± 0.07, 6.2 ± 0.09, 19.1 ± 0.07, 25 ± 0.06, 32.8 ± 0.10, 49.8 ± 0.12, 49.8 ± 0.12 and 56.9 ± 0.18% respectively with IC_50_ value of 308.4 µM, as shown in Table 4.

### Effect on PRT

**Fig. 16:**
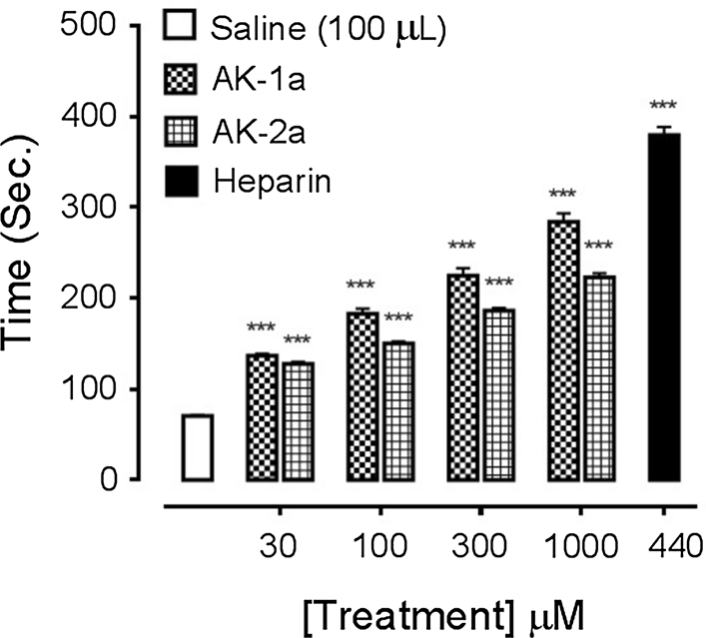
Bar chart showing increase in plasma recalcification time (PRT) caused by different concentrations of (2*E*,5*E*)-2-(4-methoxybenzylidene)-5-(4-nitrobenzylidene) cyclopentanone (AK-1a), (1*E*,4*E*)-4-(4-nitrobenzylidene)-1-(4-nitrophenyl) oct-1-en-3-one (AK-2a) and heparin. Data expressed as mean ± SEM, n = 5, ***P < 0.001 vs. saline group, one way ANOVA with post hoc Tukey’s test

At 30, 100, 300 and 1000 µM concentrations, AK-1a increased coagulation time to 137 ± 2.12, 182.8 ± 5.59, 224.6 ± 8.37 and 284 ± 9.46 s (*P *< 0.001 vs. saline group) respectively. AK-2a increased coagulation time to 128 ± 2.16, 150.6 ± 2.29, 186 ± 3.25 and 223 ± 4.47 s (*P *< 0.001 vs. saline group) respectively as shown in Fig. 16. At 440 µM concentration, heparin increased coagulation time to 379.40 ± 9.17 s (*P *< 0.001 vs. saline group).

### Effect on BT

**Fig. 17:**
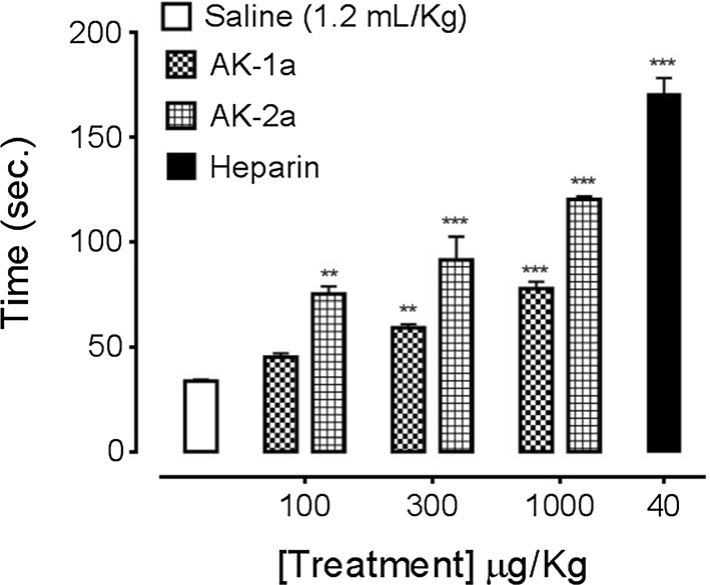
Bar chart showing increase in tail bleeding time (BT) caused by different doses of (2*E*,5*E*)-2-(4-methoxybenzylidene)-5-(4-nitrobenzylidene) cyclopentanone (AK-1a), (1*E*,4*E*)-4-(4-nitrobenzylidene)-1-(4-nitrophenyl) oct-1-en-3-one (AK-2a) and heparin in mice. Data expressed as mean ± SEM, n = 4, **P < 0.01 and ***P < 0.001 vs. saline group, one way ANOVA with post hoc Tukey’s test

At 100, 300 and 1000 µg/kg doses, AK-1a increased BT to 45.25 ± 1.75, 59.25 ± 1.65 (*P *< 0.01 vs. saline group) and 77.75 ± 3.32 s (*P *< 0.001 vs. saline group) respectively. AK-2a increased BT to 75.25 ± 3.56 (*P *< 0.01 vs. saline group), 91.50 ± 11.11 and 120.50 ± 1.44 s (*P *< 0.001 vs. saline group) respectively as shown in Fig. 17. Heparin at 40 µg/kg dose, increased BT to 170.75 ± 7.75 s (*P *< 0.001 vs. saline group).

## Discussion

In this study, we synthesized and chemically characterized two new dibenzylidene ketone derivatives. The in-silico study carried out to get an initial information about the affinity of any compound before the start of in-vivo experiment. Docking is a preliminary tool used to check the affinity of ligands to their respective protein targets. Molecular docking has an ambient role in drug discovery and development including structure based evaluation and finding target specificity and binding affinity [25]. These interactions may exist in the form of hydrogen bonds, hydrophobic interactions and Van der Waal forces. Auto Dock Vina program was used through PyRx. It uses gradient optimization method and it improves accuracy of binding mode predictions [26]. Hydrogen bonding is reported to be very significant in the formation of ligand protein complex [27]. Further we assessed affinity of ligands through E-value and number of hydrogen bonds against protein targets that influence analgesic, antiplatelet and anticoagulant effect. AK-1a and AK-2a showed highest binding affinity against PAR-1. AK-1a order of binding affinity against target proteins was found as: VKOR > COX-1 > COX-2 > F-IX > PG-I_2_ > HCR > mu receptor > GPIIb/IIa > F-II > P_2_Y_12_ > kappa receptor > F-X > delta receptor > AT-III > P2X3 > GP-VI. AK-2a order of binding affinity against target proteins was found as: COX-1 > COX-2 > HCR > mu receptor > kappa receptor > GPIIb/IIIa > PG-I_2_ > AT-III > F-IX > F-X > F-II > delta receptor > VKOR > P_2_Y_12_ > P_2_X_3_ > GPVI. We can infer that our compounds have analgesic, antiplatelet and anticoagulant actions. The analgesic activity was studied using two standard protocols i.e. acetic acid induced writhing method and hot plate assay to evaluate the peripheral and central effects of analgesia [28]. Basically writhing is an abdominal constriction caused by the release of different types of mediators after the i.p injection of acetic acid. This noxious response can be prevented by drugs which have the ability to stop the synthesis of these chemicals. The reduction in the number of writhes in treated group explains the same phenomenon of blocking the production of mediators by inhibiting COX-2 by the test compounds. Analgesic actions of AK-1a and AK-2a are proposed as inhibition of prostanoid release from cyclooxygenase involved in visceral nociception induced by acetic acid [29]. The central nociceptive effects were validated through hotplate assay [30]. AK-1a and AK-2a showed dose-dependent analgesic response, while AK-2a is found to be potent, as dose ≥ 10 mg/kg cannot be used for the analgesic activity. Significant response against acetic acid-induced writhing and hotplate assay by AK-1a and AK-2a explains central as well as peripheral activity of dibenzylidene ketone derivatives [31]. In acetic acid-induced writhing at higher dose AK-2a showed significant response, it can be further checked for anti-inflammatory response. The nociceptive behavior in the acetic acid-induced writhing test occurs due to synthesis of pain mediators including prostaglandins due to induction of COX-2 that results increased in pain sensitivity after acetic acid injection [32, 33]. Acetic acid produces nociception by releasing chemical mediators such as serotonin, histamine, prostaglandins, bradykinins and substance P due to induction of COX-2 that results in increased pain sensitivity after acetic acid injection. The acetic-induced writhing test is also sensitive to adrenoceptor agonists and opioid agonists which through appropriate receptor stimulation in the peritoneal cavity cause reduction in pain perception [34, 35]. This test involves both central and peripheral mechanisms in the early phase of the test [36]. However, hot plate test is regarded as a suitable model for the involvement of central mechanisms [37, 38]. PAR-1 activation leads to stimulation of arachidonic acid release and thrombin signaling. Arachidonic acid enhances the activation of platelet aggregation cascade [39, 40]. This can be a proposed mechanism of action for AK-1a and AK-2a as antiplatelet and anticoagulant agents. As per computational study results, both can be a potential antagonist of PAR-1 which was further validated. Curcumin analogues inhibit platelet aggregation and repress thrombosis. Dibenzylidene ketone derivatives used in this study, having ketone moiety showed significant antiplatelet and anticoagulant response [41] presence of methoxy group in AK-1a enhanced its biological activity [42]. Previous studies revealed role of curcumin derivatives as a vitamin k antagonist [43], so as these dibenzylidene ketone derivatives. Anticoagulant actions of AK-1a and AK-2a were also validated by the presence of hydrophobic groups [44].

## Conclusions

The present study reports t newly synthesized dibenzylidene ketone derivatives AK-1a and AK-2a showed high binding affinities against different protein targets involved in mediation of pain, platelet aggregation and blood coagulation process. The pharmacological investigations based on in-silico, in-vitro and in-vivo studies revealed their analgesic, antiplatelet and anticoagulant actions. These are promising findings, since the production of dibenzylidene compounds is a simple, cheap and feasible process.
